# NOTCH3 is a Prognostic Factor and Is Correlated With Immune Tolerance in Gastric Cancer

**DOI:** 10.3389/fonc.2020.574937

**Published:** 2021-01-05

**Authors:** Yuehong Cui, Qian Li, Wei Li, Yan Wang, Fang Lv, Xinying Shi, Zhaoqing Tang, Zhenbin Shen, Yingyong Hou, Henghui Zhang, Beibei Mao, Tianshu Liu

**Affiliations:** ^1^ Department of Medical Oncology, Zhongshan Hospital, Fudan University, Shanghai, China; ^2^ Medical Department, Beijing Genecast Biotechnology Co., Beijing, China; ^3^ Department of General Surgery, Zhongshan Hospital, Fudan University, Shanghai, China; ^4^ Department of Pathology, Zhongshan Hospital, Fudan University, Shanghai, China; ^5^ Center of Integrative Medicine, Beijing Ditan Hospital, Capital Medical University, Beijing, China

**Keywords:** gastric cancer, NOTCH signaling, prognosis, immune tolerance, biomarker

## Abstract

**Introduction:**

Although traditional treatments confer survival benefits to patients with gastric cancer (GC), many patients experience relapse soon after postoperative adjuvant therapy. Immune-related mechanisms play an important role in GC, and immunotherapeutic strategies are considered to be a promising direction for the treatment of GC. Thus, our study aimed to investigate the expression and prognostic significance of immune-related genes in GC.

**Methods:**

Formalin-fixed, paraffin-embedded samples were collected from 48 resectable GC patients. The transcriptome data of the tumor immune microenvironment were assessed using an immuno-oncology 395-gene panel RNA sequencing platform. The prognostic value of the 395 genes was analyzed and validated in the KM plotter and GEPIA databases. The data from The Cancer Genome Atlas (TCGA, downloaded from UCSC Xena repository) and Tumor IMmune Estimation Resource (TIMER) were used to evaluate the correlations between prognostic factors and immune signatures.

**Results:**

Among the 395 genes, NOTCH3 was identified as a good prognostic factor for GC patients. Its prognostic value was also suggested in both our GC cohort from Zhongshan Hospital and the public databases (KM plotter and GEPIA database). Mechanistically, high NOTCH3 expression correlated with a lower infiltration of activated CD8^+^ T cells and a higher infiltration of immunosuppressive cells including Tregs and M2 macrophages in the tumor microenvironment. Moreover, high NOTCH3 expression was accompanied by the increased expression of a series of immune checkpoint inhibitors, resulting in a dampened immune response. Interestingly, NOTCH3 expression had a negative association with well-documented predictive biomarkers of immune checkpoint blockade (ICB) immunotherapy, including tumor mutation burden (TMB), gene expression profiling (GEP) score and innate anti-PD-1 resistance (IPRES) signature.

**Conclusion:**

These findings uncovered a new mechanism by which NOTCH3 participates in the immune tolerance of GC, implying the potential of NOTCH3 as a therapeutic target or predictive marker for GC patients.

## Introduction

Gastric cancer (GC) is the fifth most common neoplasm in the world (1). Despite improvements in surgical techniques, radiotherapy, chemotherapy, and neoadjuvant therapy, GC remains the second leading cause of cancer death worldwide (2). Thus, identifying predictive and prognostic markers is an important step for improving current treatment approaches and extending survival.

In previous studies, researchers have established a large number of gene expression signatures for patient stratification. According to these signatures, GC patients are divided into different prognostic risk groups (3–6). Most of the signatures are mainly composed of tumor cell intrinsic genes involved in tumor proliferation and invasion. However, the progression of tumors is controlled not only by malignant cells but also by cells from the host, such as endothelial cells, stromal fibroblasts, and a variety of immune cells. Immune-related mechanisms have been increasingly recognized to play an important role in cancer progression. Cytotoxic T cells, regulatory T cells (Tregs), dendritic cells (DCs), tumor-associated macrophages, and mesenchymal stem cells have been reported to be closely associated with the clinical outcomes of patients with solid tumors (7–9). Three subtypes of tumor microenvironment (TME) cell infiltration showed different survival times in GC. TME cluster-A, which was characterized by increases in the infiltration of immunosuppressive cells, had the poorest outcome. In contrast, TME cluster-C, which showed significant increases in the infiltration of CD8^+^ T cells, M1 macrophages, and activated memory CD4^+^ T cells, had a favorable outcome (10).

In addition, immunotherapeutic strategies, especially immune checkpoint blockade (ICB), have become the standard care in various types of cancer. However, only a small proportion of GC patients respond to current ICB therapy. Given the observation that the immune-related TME is essential for the prognosis and efficacy of chemotherapy and immunotherapy (11, 12) in other cancer types, it is worth investigating tumor-immune interactions and identifying novel potential prognostic and therapeutic targets in GC patients.

Herein, transcriptome data of the tumor immune microenvironment from 48 stage III GC patients were assessed using an immuno-oncology (IO) panel RNA sequencing platform. Analysis of the prognostic value of these genes revealed that high mRNA levels of NOTCH3 were associated with poor prognosis in GC, which was validated in two public databases. Importantly, further investigation revealed the correlation of NOTCH3 with tumor-infiltrating immune cells, immune checkpoint gene expression, and well-established biomarkers of ICB therapy. These findings uncovered a new mechanism by which NOTCH3 participates in the progression and immune tolerance of GC, implying the potential of NOTCH3 as a therapeutic target or predictive marker for GC patients.

## Materials and Methods

### Patient Information and Sample Collection

The formalin-fixed, paraffin-embedded (FFPE) samples of 48 GC patients after radical resection were retrieved from the tissue achieve of Zhongshan Hospital (Shanghai, China) in 2015, and the last follow-up date was April 2019. All of the patients were stage III. Clinically recorded information, such as age, sex, tumor histology, and pathologic stage, were collected and the detailed information was shown in **Table 1**. The institutional review board of Zhongshan Hospital gave explicit approval for the study, and all samples were obtained upon informed consent under an institutional protocol for tissue collection. There were 35 male and 13 female patients. The median age was 62.5 ± 9.82 years, and the median disease-free survival (DFS) was 14.85 ± 0.11 months. Sections from the FFPE blocks were cut and stained with hematoxylin and eosin. Tumor tissues were confirmed by a qualified pathologist. Additional serial sections were cut for RNA extraction.

**Table 1:** Baseline characteristics of patients with gastric cancer from Zhongshan cohort (N = 48).

Factors		Cohort (N = 48)
**Gender**		
	Male	35 (73%)
	Female	13 (27%)
**Lauren classification**		
	Diffusal type	18 (38%)
	Non-diffusal type	30 (62%)
**T stage**		
	T4	33 (69%)
	T3	13 (27%)
	T2	2 (4%)
**N stage**		
	N3	33 (69%)
	N2	14 (27%)
	N1	1 (2%)
**Perineural invasion**		
	Positive	38 (79%)
	Negative	10 (21%)
**Vascular cancerous embolus**		
	Positive	41 (85%)
	Negative	7 (15%)

### RNA IO Profiling

The RNA IO profiling platform (Genecast Biotechnology, Beijing, China) is a unique 395-plex gene expression panel that quantifies 395 IO-related genes in human solid cancers that mainly fall into the following categories: tumor markers and essential signaling pathways, tumor-specific antigens, immunological response, infiltrating immune cells, and housekeeping (HK) genes. In brief, RNA was extracted from FFPE tissues by means of the truXTRAC™ FFPE RNA Kit (Covaris, Inc., Woburn, MA). After purification, the RNA yield was quantified using a Qubit™ RNA HS Assay Kit (Thermo Fisher Scientific, Waltham, MA). Then, 10 ng of RNA was reverse transcribed into cDNA, and targets were amplified with the primer pool targeting 395 genes. Barcode adapters were ligated to partially digested amplicons. Purified libraries were quantified *via* an Agilent™ 2100 Bioanalyzer (Agilent, Santa Clara, CA) and then pooled in equal molar amounts prior to enrichment and template preparation using the Ion Chef™ system (Thermo Fisher Scientific, Waltham, MA). For each sample, 200 bp sequencing was performed on the Ion S5 530 chip (Thermo Fisher Scientific, Waltham, MA) to obtain 1–2 M reads. The absolute digital gene expression counts of all samples in the same run were automatically generated in the in-house bioinformatics pipeline. Only sequencing data meeting the quality control (QC) criteria for mapped reads, on-target reads and mean reads were included in the study.

Gene expression normalization was performed as described previously (13). A baseline expression profile for 10 HK genes was established based on the average reads per million (RPM) counts. Each HK gene background-subtracted read was compared against the RPM profile from that internal control sample, which then gave rise to a fold-change ratio for each HK gene: ratio of HK = absolute read count of HK/RPM profile of HK. Then, the normalization ratio for the particular sample was calculated using the median value of all HK ratios. The normalization ratio equals the median (all HK ratios). Next, the nRPM of all genes (G) of the specific sample (S) (nRPM(S,G)) was calculated as:

$$nRPM\;(S,G)=\frac{Background-Subtracted\;\text{Read}\;\;Count\;(S,G)}{Normalization\;Ratio\;(S,G)}$$

### Data Acquisition

The TCGA gene expression data of stomach adenocarcinoma (STAD), colon adenocarcinoma (COAD), esophageal carcinoma (ESCA), liver hepatocellular carcinoma (LIHC), and pancreatic adenocarcinoma (PAAD) were acquired *via* the UCSC Xena repository. The scores of 28 immune cell types and functions in these samples were inferred from the gene expression data by single-sample gene set enrichment analysis (ssGSEA) (14). The abundances of M1 macrophages and M2 macrophages were estimated by CIBERSORT (15).

### Statistical Analysis

For comparisons between two groups, statistical significance for normally distributed variables was estimated by unpaired Student’s t test, and nonnormally distributed variables were analyzed by the Mann–Whitney U test. Correlation coefficients were computed by Spearman analyses. The Kaplan–Meier method was used to generate survival curves for the subgroups in each data set, and the log-rank (Mantel–Cox) test was used to determine the statistical significance of differences. The hazard ratios for univariate analyses were calculated using a univariate Cox proportional hazards regression model. A multivariate Cox regression model was used to determine independent prognostic factors by SPSS19.

## Results

### Prognostic Potential of NOTCH3 in Gastric Cancer

**Figure 1:**
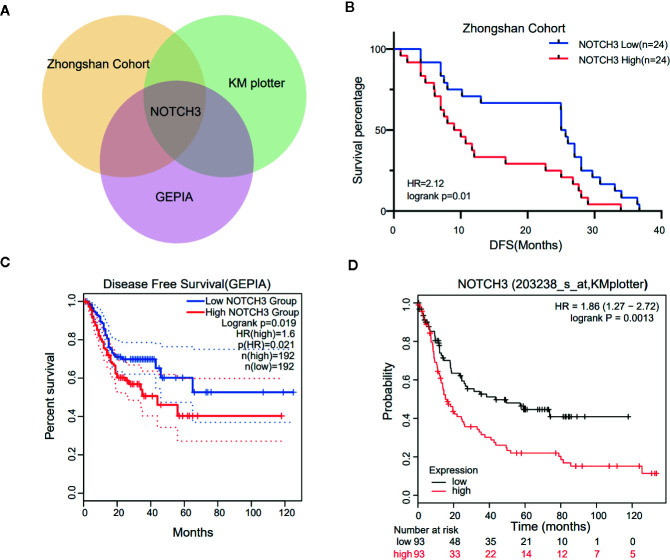
Kaplan-Meier survival curves illustrate the prognostic value of NOTCH3 expression in gastric cancer (GC) patients. **(A)** Venn diagram showed the genes whose prognostic value could be validated in three cohorts. **(B)** Survival curves of disease-free survival (DFS) in GC cohort from Zhongshan hospital. **(C)** Survival curves of DFS in stomach adenocarcinoma (STAD) cohort from GEPIA database. **(D)** Survival curves of first progression (FP) in GC cohort from KM-plotter database.

To maintain sufficient group sizes for the analysis, 48 resectable patients from Zhongshan Hospital were divided based on the median expression level of 395 immune-related genes. Using univariate Cox regression analysis, we identified 73 genes significantly associated with the DFS of patients (the 73 genes are listed in **Supplemental Table 1**). Then, we examined the influence of the above 73 genes’ expression on the prognosis of GC in the GEPIA and KM Plotter databases. We observed that among the 73 genes, only NOTCH3 was significantly related to patient survival in both the GEPIA (STAD) and KM Plotter (GC) cohorts when we set the median expression of NOTCH3 as a cutoff to stratify patients (**Figure 1A**, log-rank test *P*<0.05). In accordance with the Zhongshan cohort (**Figure 1B**), patients with high NOTCH3 expression had a shorter DFS or first progression (FP) than patients with low NOTCH3 expression (**Figures 1C, D**, log-rank test *P*<0.05). Furthermore, multivariate Cox regression analysis incorporating the clinical features of the 48 GC patients in the Zhongshan cohort revealed that NOTCH3 expression was an independent prognostic factor for GC patients (**Table 2**).

**Table 2:** Univariate and multivariable Cox regression analyses for disease-free survival (DFS) time of 48 patients with gastric cancer from Zhongshan Cohort.

Variables	Univariate analysis		Multivariate analysis	
	HR (95%CI)	*P* value	HR (95%CI)	*P* value
**Gender**		0.055		0.073
Male	1		1	
Female	1.951 (0.987–3.859)		2.157(0.932–4.990)	
**Vascular tumor thrombus**		0.723		0.812
Negative	1		1	
Positive	0.863 (0.383–1.947)		0.883(0.317–2.462)	
**Perineuronal invasion**		0.12		0.551
Negative	1		1	
Positive	1.880 (0.848–4.165)		1.302(0.547–3.102)	
**Lauren classification**		**0.049**		0.245
Non-Diffused	1		1	
Diffused	1.881 (1.004–3.524)		1.512(0.753–3.039)	
**T stage**		0.518		0.792
T2/3	1		1	
T4	1.227 (0.660–2.280)		1.115(0.497–2.502)	
**N stage**		0.833		0.210
N1/2	1		1	
N3	1,069 (0.577–1.981)		1.651(0.754–3.613)	
**NOTCH3**		**0.014**		**0.014**
Low	1		1	
High	2.12 (1.165–3.858)		2.12 (1.165–3.858)	

Bold values represented p < 0.05.

Given the observation that NOTCH3 has prognostic value in GC, the RNA expression data in The Cancer Genome Atlas (TCGA) database were also used to analyze the prognostic potential of NOTCH3 in other gastrointestinal (GI) cancers, including LIHC, ESCA, COAD, and PAAD. The optimal cutoff values of NOTCH3 expression to determine the prognosis of patients in the TCGA cohort were calculated on the basis of the prognostic significance using X-Tile software (16). High NOTCH3 expression levels were associated with a poor prognosis of DFS and progression-free survival (PFS) in COAD, PAAD and ESCA. Moreover, high NOTCH3 expression was also correlated with shorter overall survival (OS) in COAD patients. However, NOTCH3 expression has no prognostic value for patients with liver cancer (**Figure S1**). Taken together, these results suggest the prognostic value of NOTCH3 expression in GC and other GI cancers.

### NOTCH3 Expression Is Correlated With Immune Infiltration in Gastric Cancer

**Figure 2:**
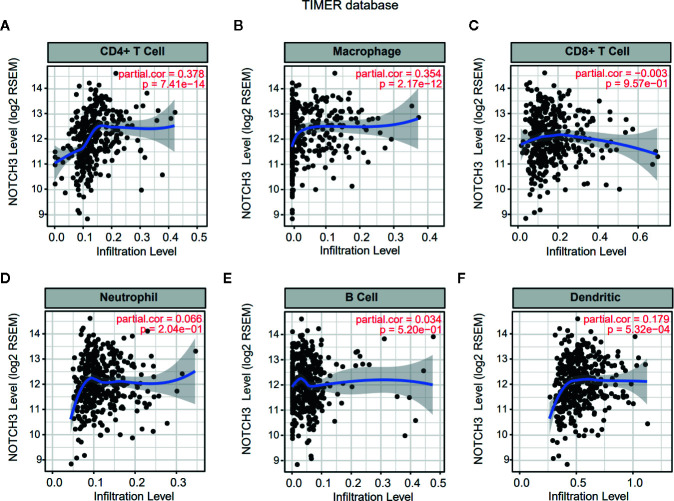
Correlation of NOTCH3 expression with immune infiltration in gastric cancer (GC) patients from Tumor IMmune Estimation Resource (TIMER) database. NOTCH3 expression has a positive correlation with infiltration of CD4^+^ T cells **(A)** and macrophages **(B)**, but a very weak correlation with CD8^+^ T cells **(C)**, neutrophils **(D)**, B cells **(E)**, dendritic cells **(F)** in stomach adenocarcinoma (STAD) (n=414) patients.

Lymphocyte infiltration in the TME is associated with the clinical prognosis of many kinds of cancers, including GC, melanoma, urothelial cancer, lung cancer, and breast cancer (17–20). The NOTCH signaling pathway has also been reported to be involved in the proliferation, differentiation and activation of lymphocytes in the thymus or peripheral lymphoid organs (21, 22). However, less is known about whether NOTCH signaling has an impact on the infiltration of lymphocytes in the TME. Therefore, we aimed to explore the correlation of NOTCH3 expression with immune cell infiltration in GI cancers. First, the association of tumor-infiltrating immune cell (TIIC) abundance with NOTCH3 expression was estimated in GC from the Tumor IMmune Estimation Resource (TIMER) database. Interestingly, the expression of NOTCH3 was positively correlated with the infiltration of CD4^+^ T cells and macrophages in STAD patients (**Figures 2A, B**). In contrast, NOTCH3 expression had a poor correlation with the enrichment of B cells, CD8^+^ T cells, neutrophils and DCs (**Figures 2C–F**). As expected, the positive association of NOTCH3 expression with CD4^+^ T cells and macrophages was also observed in COAD, LIHC and PAAD (**Figure S2**).

**Figure 3:**
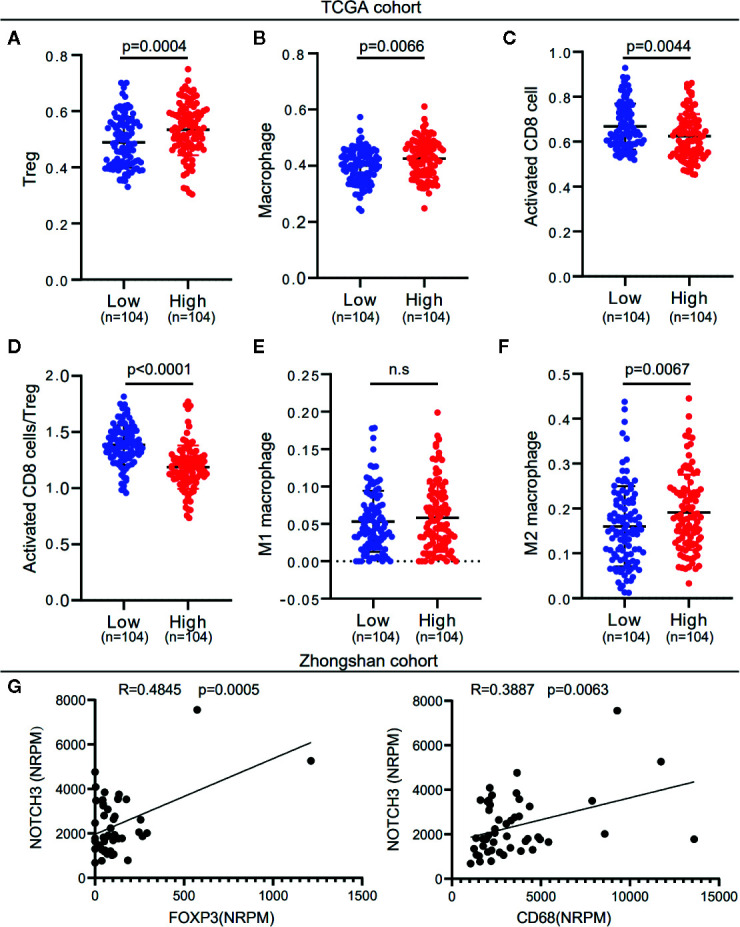
NOTCH3 upregulation is associated with increased immune suppressive cell enrichment in tumor microenvironment (TME) of gastric cancer (GC) patients. **(A–C)** Tregs **(A)**, macrophages **(B)**, and activated CD8^+^ cells **(C)** abundance was quantified by ssGSEA. **(D)** The boxplot showed the ratio of activated CD8^+^ T cell *versus* Tregs in indicated groups. **(E, F)** Comparison of M1 **(E)** and M2 **(F)** macrophage fractions between NOTCH3 High and Low group. The analyzed data of **(A–F)** was obtained from The Cancer Genome Atlas (TCGA) stomach adenocarcinoma (STAD) cohort. The percentages of M1 and M2 macrophages in each tumor sample were quantified by CIBERSORT. **(G)** The NOTCH3 expression had positive correlations with FOXP3 (left) and CD68 (right) in patients from Zhongshan cohort.

To investigate whether NOTCH3 expression has an impact on lymphocyte infiltration with further evidence at the level of sub-immune cell types, we downloaded the RNA-expression data of 414 GC (STAD) patients from the TCGA database. ssGSEA was applied to calculate the enrichment of 28 immune cell types. We defined the patients with NOTCH3 expression higher than the 75th percentile as the “NOTCH3 High group” (n=104) and patients with NOTCH3 expression lower than the 25th percentile as the “NOTCH3 Low group” (n=104). Notably, most of the immunosuppressive cells, such as Tregs, macrophages, mast cells and myeloid-derived suppressor cells (MDSCs), exhibited significantly higher enrichment levels in the NOTCH3 High group (**Figure S3**, **Figures 3A, B**). Surprisingly, activated CD8^+^ T cells showed a reduction in the NOTCH3 High group (**Figure 3C**). Thus, the ratio of activated CD8^+^ T cells to Tregs was elevated in the NOTCH3 Low group (**Figure 3D**), suggesting a graver imbalance of acquired immunity in the TME of patients with higher expression levels of NOTCH3.

To further determine whether NOTCH3 expression is correlated with M1 or M2 macrophages, we used the CIBERSORT deconvolution method to calculate the enrichment of M1 and M2 macrophages. As shown in **Figures 3E, F**, macrophages in the GC TME were mainly composed of M2 macrophages. M2 but not M1 macrophages were more abundant in the NOTCH3 High group. Concordantly, in the 48 GC patients in this study, NOTCH3 expression had a significant positive correlation with the expression of FOXP3 and CD68, which are the biomarkers of Tregs and macrophages, respectively (**Figure 3G**). Therefore, our analysis indicated that high NOTCH3 expression is positively correlated with immunosuppressive cells and inversely correlated with cytotoxic T cells, which contributes to the shorter survival of GC patients.

### Association of NOTCH3 Expression With Immune Checkpoint Inhibitors

Tumor cells escape immune surveillance and progress through different mechanisms, such as the overexpression of inhibitory immune checkpoint molecules that impair the antitumor immune response (23, 24). In this study, we first analyzed the association of NOTCH3 expression with a series of immune checkpoint inhibitors in the TCGA database. As shown in **Figure S4A**, multiple inhibitory checkpoint molecules, including CD274, CTLA4, CD276, HAVCR2 and CD200, had positive correlations with NOTCH3 mRNA levels in GC and other GI cancers.

**Figure 4:**
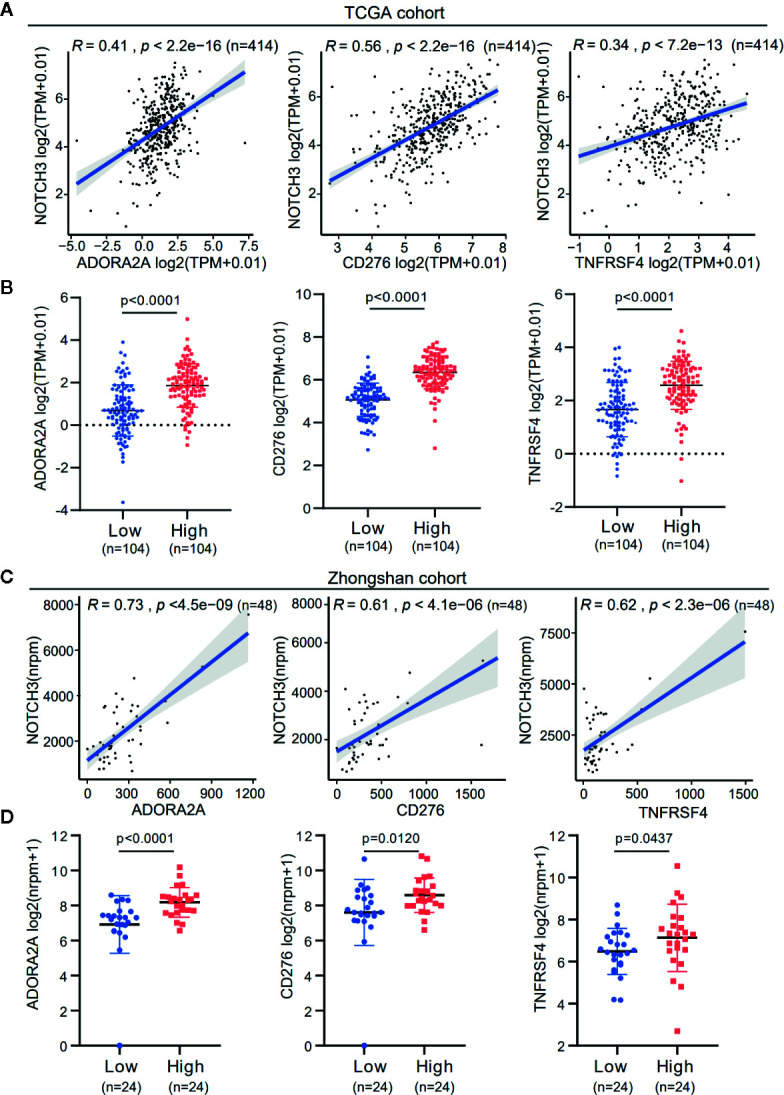
The expression association of NOTCH3 with immune checkpoint inhibitors in gastric cancer (GC) patients. **(A)** Association analysis between expression of NOTCH3 and ADORA2A, CD276, TNFRSF4 in The Cancer Genome Atlas (TCGA) stomach adenocarcinoma (STAD) database. **(B)** Boxplot distributions between groups with different NOTCH3 expression for ADORA2A, CD276, TNFRSF4 according to TCGA STAD database. **(C)** In the cohort from Zhongshan Hospital, linear regression curves displayed the correlation between expression of NOTCH3 and ADORA2A, CD276, TNFRSF4. **(D)** The boxplot illustrated the distribution of ADORA2A, CD276, TNFRSF4 expression in NOTCH3 differentially expressed patients from Zhongshan cohort.

Additionally, the expression of immune checkpoint genes, as mentioned above, was significantly higher in the NOTCH3 High group (n=104) than in the NOTCH3 Low group (n=104) in GC patients (**Figure S4B**). It is worth mentioning that the expression of three genes, ADORA2A, CD276 (B7-H3) and TNFRSF4, correlated with NOTCH3 in the TCGA STAD database (**Figures 4A, B**). This correlation was validated in the Zhongshan cohort of 48 patients (**Figure 4C**). The expression of these three genes was also significantly reduced in the NOTCH3 Low group when the 48 patients were stratified according to the median expression of NOTCH3 as the cutoff (**Figure 4D**). These data suggest that multiple immune checkpoint pathways, not only the PD-1/PD-L1 axis, cause immune tolerance and escape in patients with high NOTCH3 expression and result in poor outcomes in GC patients.

### Relationship Between NOTCH3 Expression and the Currently Adopted Predictive Biomarkers for Immune Checkpoint Blockade Therapy

**Figure 5:**
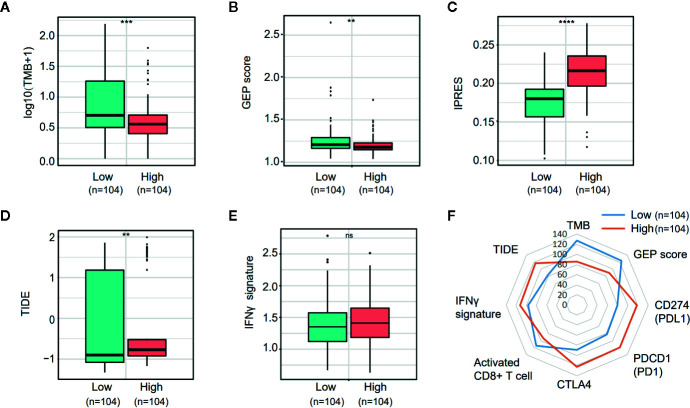
Comparison of the biomarkers for immune checkpoint blockade therapy between NOTCH3 High and Low group in The Cancer Genome Atlas (TCGA) database. **(A, B)** The boxplot indicated tumor mutation burden (TMB) **(A)** and gene expression profiling (GEP) score **(B)** were reduced in NOTCH3 High group comparing with NOTCH3 Low group. **(C, D)** The boxplot illustrated the higher innate anti-PD-1 resistance (IPRES) **(C)** and Tumor Immune Dysfunction and Exclusion (TIDE) score **(D)** in NOTCH3 High group. **(E)** The IFNγ signature showed no difference in NOTCH3 High and Low groups. **(F)** The radar chart summarized the association between NOTOCH3 expression and the currently-adopted predictive biomarkers for immune checkpoint blockade therapy. Each point indicates the median rank of predictive biomarkers in NOTCH3 High and Low groups of gastric cancer (GC) patients.

PD-L1 (CD274) and CTLA4 are two well-studied immune checkpoints targeted for the treatment of patients with cancer (25–28). Numerous studies revealed that in addition to PD-L1 expression stained by IHC, biomarkers such as the density of tumor-infiltrating lymphocytes (TIL), tumor mutation burden (TMB), mismatch repair (MMR) deficiency, IFN-γ signature, and the 18-gene T-cell–inflamed gene expression profiling (GEP) score have been associated with the treatment effect of anti-PD-1/PD-L1 therapy (29–32). Given the observation that NOTCH3 expression had an impact on the infiltration of lymphocytes (**Figure 3**) and checkpoint inhibitor expression (CD274, CTLA4) (**Figure S4B**), we wondered whether NOTCH3 expression has an effect on other features predictive of ICB therapy. As shown in **Figures 5A, B**, in the TCGA STAD cohort, both TMB and GEP score, which have been demonstrated to be positively correlated with the prognosis of patients receiving pembrolizumab treatment (29, 32), were lower in the NOTCH3 High-expressed group.

Conversely, the innate PD-1 resistance (IPRES) gene signature (33) for the prediction of resistance to PD-L1 or CTLA-4 blockade was much higher in the NOTCH3 High group (**Figure 5C**). Similarly, patients with high NOTCH3 expression had a higher Tumor Immune Dysfunction and Exclusion (TIDE) signature (34), implying the failure of ICB therapy in the NOTCH3 High group (**Figure 5D**). Regarding the IFN-γ signature (35), there was no significant difference between the two groups (**Figure 5E**). Overall, despite the differential expression of PD-1, PD-L1 and CTLA4 at the mRNA level (**Figure S4B**, **Figure 5F**), the impact of NOTCH3 expression on the other biomarkers of ICB therapy can be inferred as patients with high NOTCH3 expression may be more intrinsically resistant to ICB therapy.

## Discussion

Immune cells and cytokines in the TME have a crucial influence on the occurrence and development of malignant cancers. In this study, we conducted immune-related RNA IO panel sequencing on 48 GC patients. Combining the data from two public databases, we identified NOTCH3 as a prognostic factor in GC and other GI cancers. We also delineated the molecular properties of immunogenicity associated with NOTCH3 expression, providing a potential target or predictive factor for GC immunotherapy.

The NOTCH signaling pathway is essential for cell fate and organogenesis. Accumulated evidence indicates that members of the NOTCH family play a central role in tumor malignancies, and they might serve as biomarkers for the diagnosis and prognosis of cancer (36–38). There are four NOTCH receptors (NOTCH1-4) in mammals. It has been reported that high NOTCH1 and NOTCH3 expression levels are related to poor OS rates in cancer (39, 40). However, low NOTCH2 expression levels are related to poor outcomes in cancer (41). To date, the contribution of NOTCH3 to GC development has not been fully elucidated. In our study, NOTCH3 was verified to be associated with the survival of GC patients using our cohort and public databases, and the prognostic value of NOTCH3 in GC patients was confirmed.

Increasing evidence has shown that the NOTCH pathway facilitates tumorigenesis and progression by initiating the transcription of target genes involved in tumor cell proliferation, differentiation, invasion and angiogenesis (42). It is also recognized that the progression of tumors is not only controlled by the intrinsic changes in cancerous cells but also dependent on the activity of nonmalignant cells in the TME, especially lymphocyte infiltration and activation (43). By the CIBERSORT analysis of TCGA RNA data, we found that activated CD8^+^ T cells were enriched in the NOTCH3 Low group (**Figure 3C**). At the same time, NOTCH3 expression was positively correlated with genes of immune checkpoint inhibitors, including ADORA2A (A2a receptor) and CD276 (B7-H3) (**Figure 4**). Accordingly, Yu and his colleagues reported that NOTCH signaling plays a negative role in regulating the antitumor activity of CD8^+^ T cells by upregulating the expression of PD-1 (44). Therefore, complementary to the lower infiltration of CD8^+^ T cells, these checkpoint inhibitors form a counter-regulatory mechanism in cancer cells putatively driven by the high expression of NOTCH3, indicating a new mechanism of NOTCH signaling for facilitating immune escape. Cytotoxic T cells play a crucial role in determining the clinical outcomes of patients in various types of cancer. Jung Soo Lee and his colleagues found that the high expression of TILs, mainly CD8^+^ T cells, may be a potential prognostic biomarker in patients with GC (45). It was reported that GC patients with high-density groups of CD3, CD8, and CD45RO cells had significantly longer survival times than those with low-density groups of these cells (46). Thus, the high expression of NOTCH3 with low activity of cytotoxic adaptive immunity probably contributes to the poor prognosis of GC patients.

In contrast to CD8^+^ T cells, Tregs are immunosuppressive cells in the TME. Through IL-10 and IL-35 cytokine production, tumor-infiltrating Tregs help tumor cells escape immune surveillance (47, 48). In addition to Tregs, M2 macrophages are another important immunosuppressive innate immune cell that is abundant in the TME. M2 macrophages promote tumor growth, progression, invasion and metastasis by producing anti-inflammatory cytokines, such as IL-10 and TGF-β (49, 50). Both Tregs and M2 macrophages protect tumor cells from discovery and elimination by the normal immune system, resulting in poor outcomes in GC (51, 52). The important role of NOTCH signaling in regulating these cell types has been confirmed by different researchers (53–56). Notwithstanding, most of the studies were performed in the context of mouse models or isolated cultured cells. Our results from the analysis of the TCGA database showed that high NOTCH3 expression was correlated with a high content of immunosuppressive Tregs and M2 macrophages (**Figures 3A, F**). This finding was also supported by the positive correlation between NOTCH3 and FOXP3 or CD68 expression in Zhongshan cohort (**Figure 3G**). These conclusions were drawn based on the results from the clinical specimens of cancer patients, and the correlation between NOTCH signaling and the infiltration of these immunosuppressive lymphocytes in the TME was verified.

The suppression of immune checkpoints with monoclonal antibodies blocking PD-L1 and PD1 has been proven to be an unprecedented anticancer therapy in different kinds of cancers (57–59). However, the responses occur in only a small fraction of patients. In particular, in GC, the overall response rate of anti-PD-1/PD-L1 treatment was only approximately 12%, and some patients were prone to developing hyperprogressive disease (HPD) (57, 60). Several biomarkers, including TMB, PD-1/PD-L1 expression, microsatellite instability (MSI) status and GEP score, have been well established for predicting the benefits of ICB. To date, in GC patients, no clinically validated biomarkers could completely distinguish responders from nonresponders despite the T-cell–inflamed GEP score (57). Intriguingly, patients with high NOTCH3 expression had significantly lower TMB and GEP scores than those with low NOTCH3 expression. In contrast, the IPRES signature, which represents the resistance to ICB therapy, and the TIDE score, which describes the dysfunction and exhaustion of CD8^+^ T cells, were both higher in patients with high NOTCH3 expression (**Figure 5**). Therefore, NOTCH expression has the potential to predict the efficiency of ICB therapy. There are few studies directly linking NOTCH signaling with ICB therapy in addition to Donghai Xiong’s finding that in one patient with esophageal squamous cell carcinoma (ESCC) who developed HPD after anti-PD-1 immunotherapy, somatic mutations of NOTCH4 were not present in the pretherapy tumor (61). Although our findings have important implications for the treatment of GC patients with different NOTCH3 expression levels, whether GC patients with low NOTCH3 expression could benefit from ICB therapy needs to be evaluated directly in clinical practice.

In conclusion, our research provided convincing data on the prognostic value of NOTCH3 expression in GC patients. Mechanistically, we found that the effect of NOTCH signaling on the infiltration and activity of immune cells, especially CD8^+^ T cells, Tregs and M2 macrophages, in the TME is involved in its contribution to the prognosis of GC. In particular, NOTCH signaling was correlated with the well-documented predictive biomarkers of immunotherapy, which would shed new light on the discovery of potential predictive biomarkers for GC patients.

## Data Availability Statement

The original contributions presented in the study are publicly available. This data can be found here: https://db.cngb.org/cnsa/project/CNP0001464/reviewlink/, accession number CNP0001464.

## Ethics Statements

The studies involving human participants were reviewed and approved by The institutional review board of Zhongshan Hospital gave explicit approval for the study. The patients/participants provided their written informed consent to participate in this study.

## Author Contributions

YC wrote the first draft of manuscript. TL and HZ contributed to the conception and design of the research. QL, XS, and FL contributed to the experiment and analysis of the data. BM contributed to the analysis and interpretation of the data. All authors contributed to the article and approved the submitted version.

## Funding

This research was supported by the ① Study on the prevention and control of major chronic non-infectious diseases, National Key Research and Development Plan of China, 2017YFC1308900, and ② The Science and Technology Commission of Shanghai Municipality, 19ZR1409500.

## Conflict of Interest

The authors FL, XS, and BM were employed by Beijing Genecast Biotechnology Co.

The remaining authors declare that the research was conducted in the absence of any commercial or financial relationships that could be construed as a potential conflict of interest.

## Abbreviations

COAD, colon adenocarcinoma; DCs, dendritic cells; DFS, disease-free survival; ESCA, esophageal carcinoma; GC, gastric cancer; GEP, gene expression profiling; ICB, immune checkpoint blockade; IO, immuno-oncology; IPRES, innate anti-PD-1 resistance; LIHC, liver hepatocellular carcinoma; OS, overall survival; PAAD, pancreatic adenocarcinoma; PFS, progression-free survival; ssGSEA, single-sample gene set enrichment analysis; STAD, stomach adenocarcinoma; TCGA, The Cancer Genome Atlas; TIIC, tumor-infiltrating immune cell; TIMER, Tumor Immune Estimation Resource; TMB, tumor mutation burden; TME, tumor microenvironment; Tregs, regulatory T cells.
