# Supplementary figures and images for: NOTCH3 is a Prognostic Factor and Is Correlated With Immune Tolerance in Gastric Cancer
Source: Front Oncol. 2021 Jan 5;10:574937. doi: 10.3389/fonc.2020.574937 (PMC7814877; doi:10.3389/fonc.2020.574937)

Figure.S1

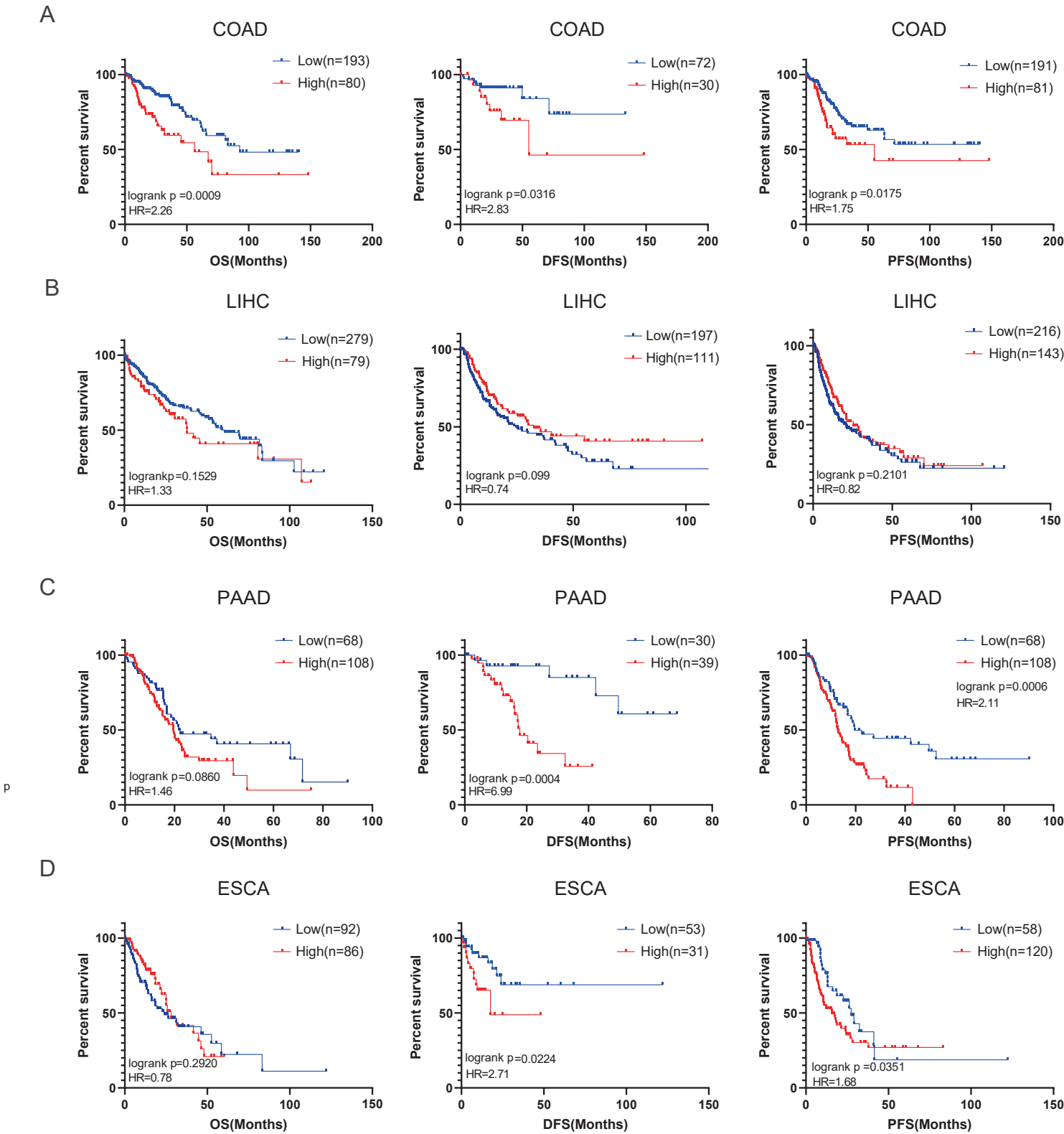

Supplement: Supplementary file 2 [file Image_1.pdf]

Figure.S2

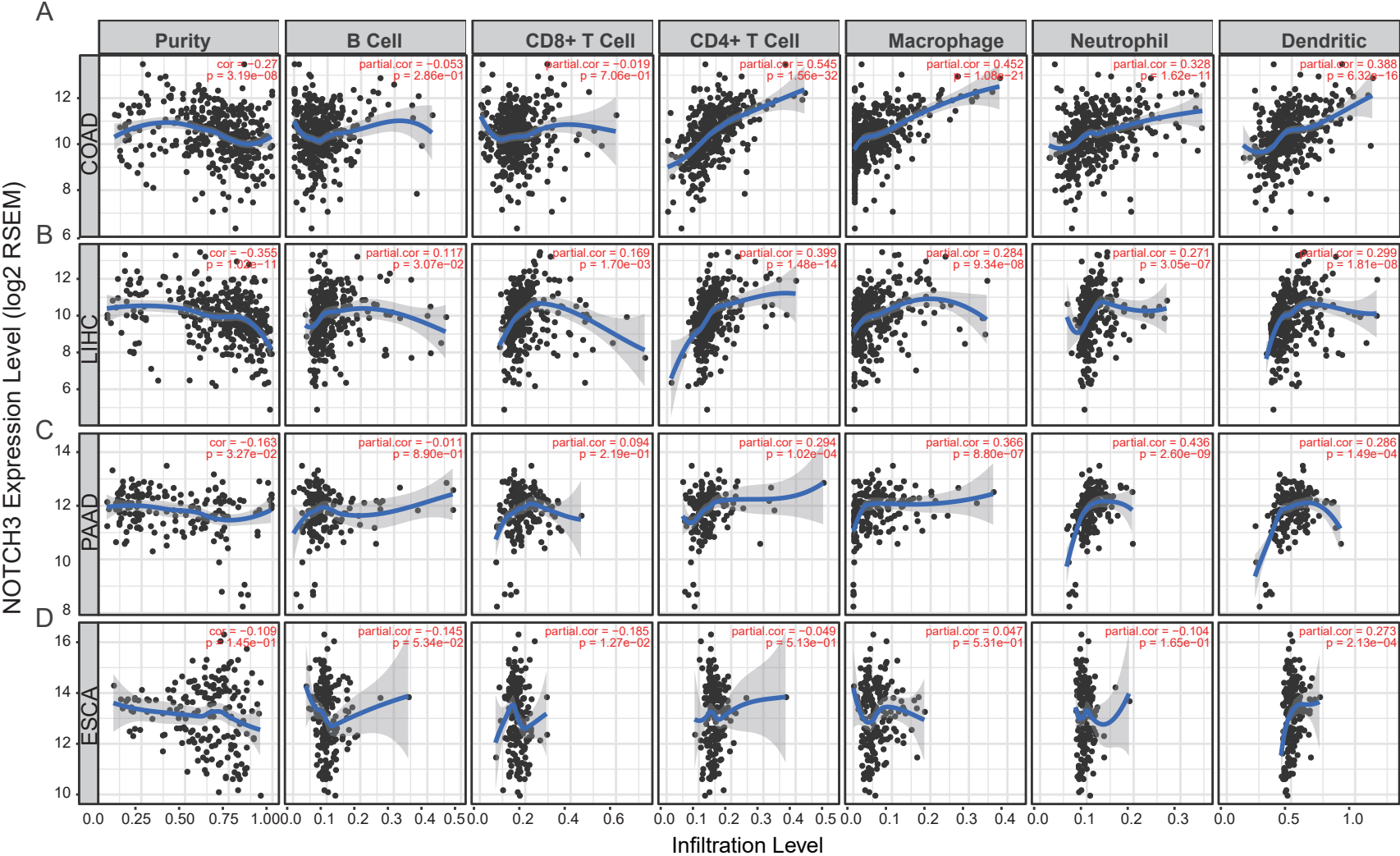

Supplement: Supplementary file 3 [file Image_2.pdf]

Figure.S3

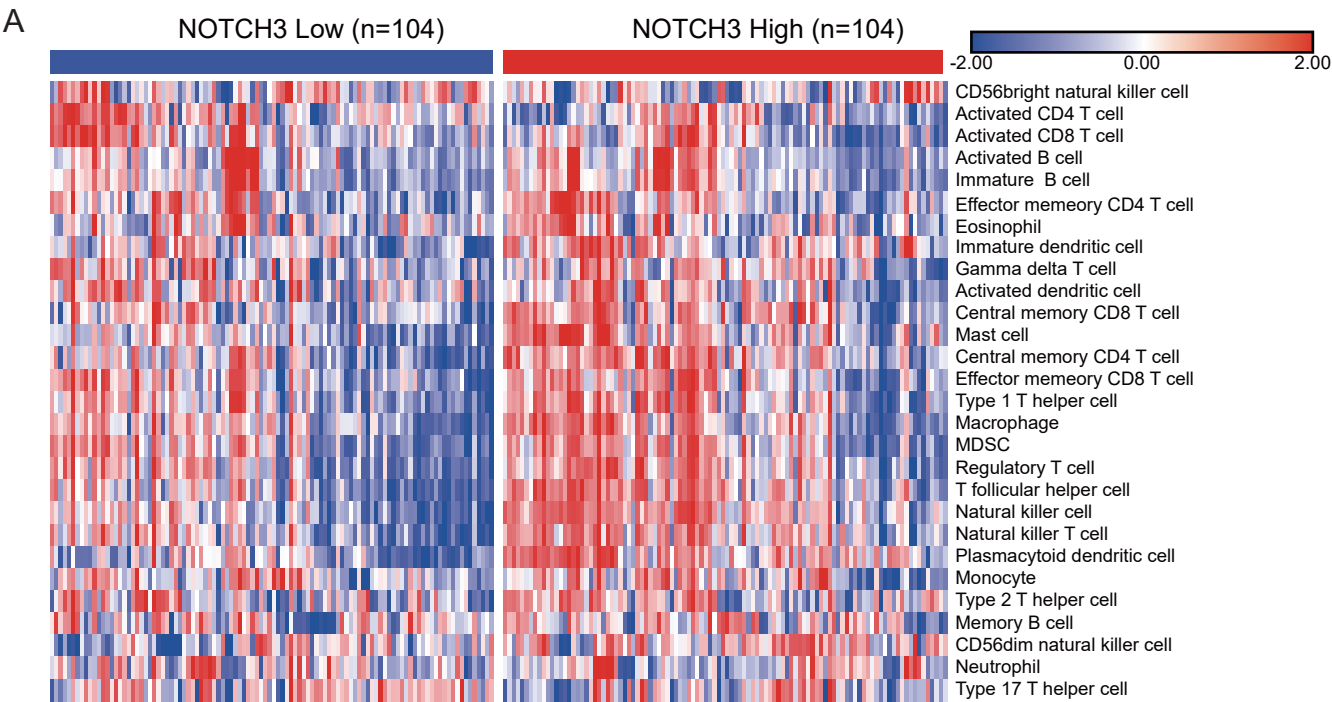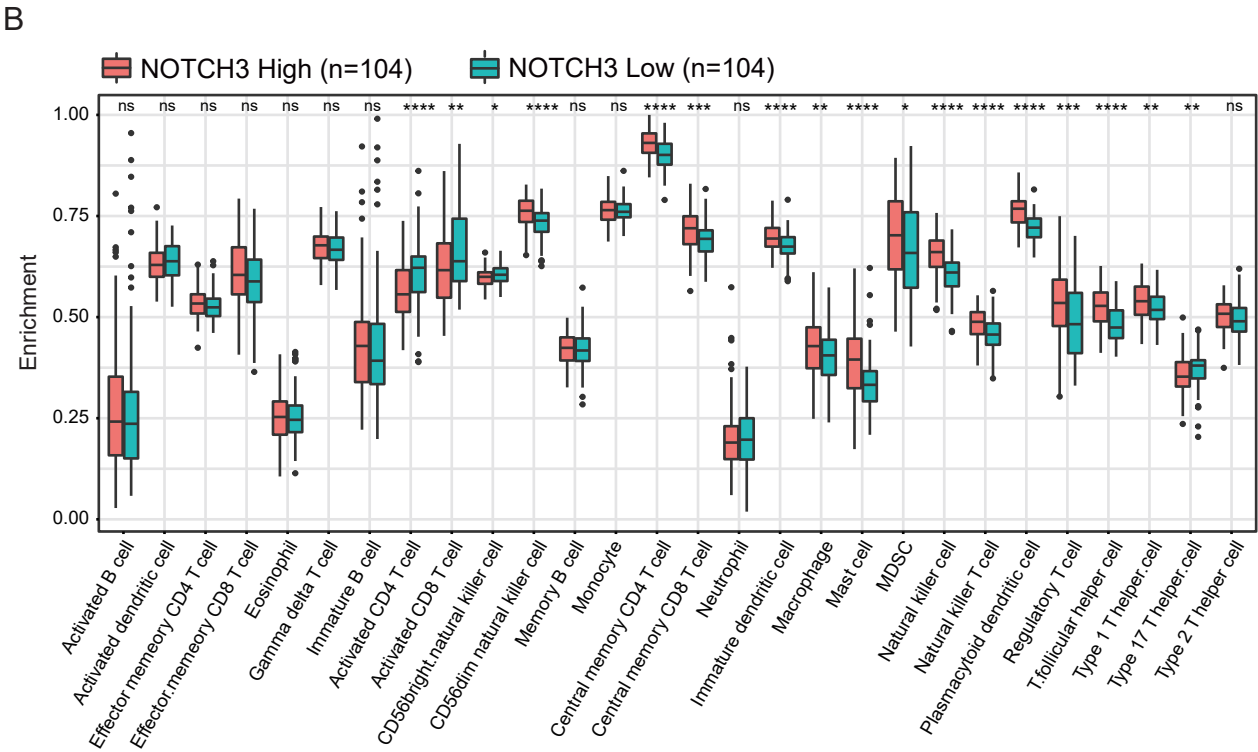

Supplement: Supplementary file 4 [file Image_3.pdf]

Figure.S4

A

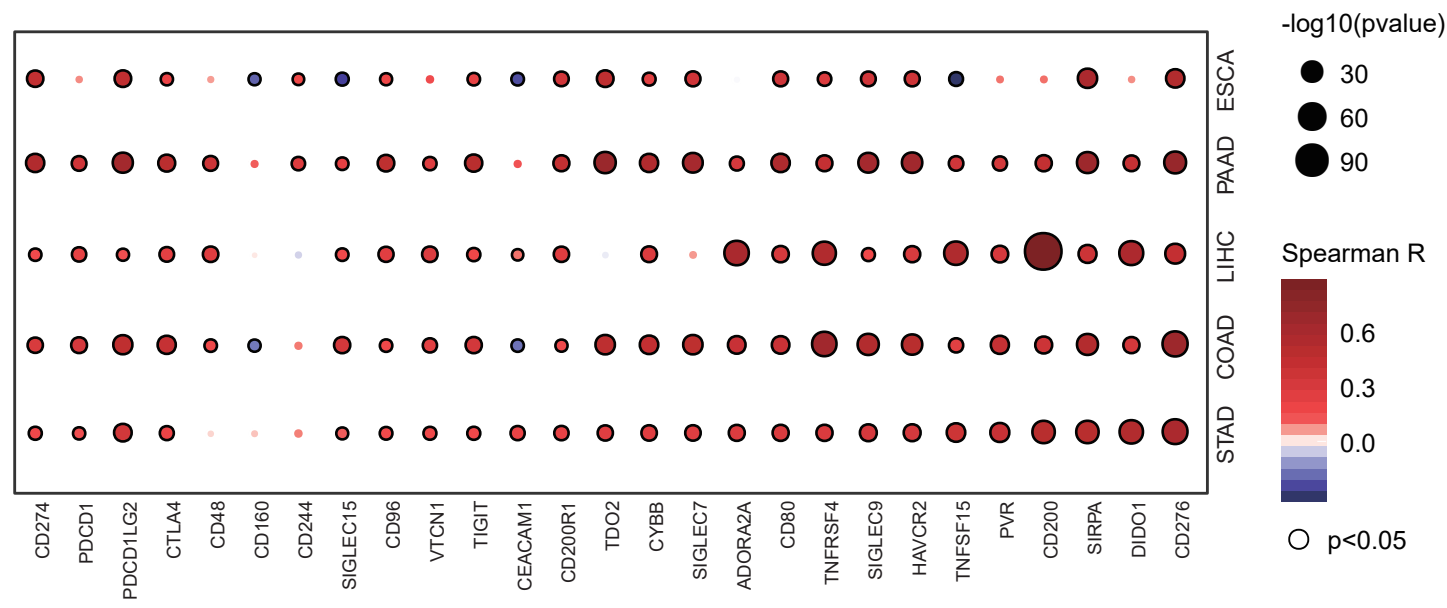

B

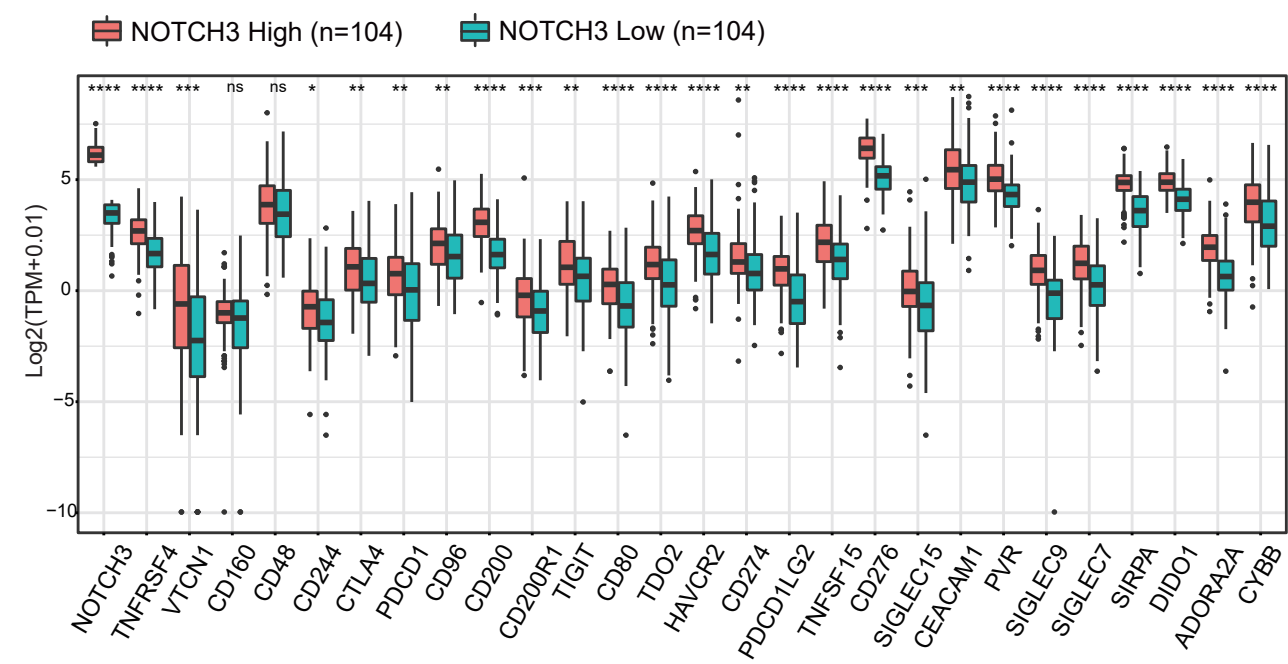

Supplement: Supplementary file 5 [file Image_4.pdf]
